# Impact of HLA locus mismatch on peripheral blood allogeneic hematopoietic stem cell transplantation from unrelated donors using an ATG-based GVHD prophylaxis strategy

**DOI:** 10.1007/s44313-025-00082-6

**Published:** 2025-06-03

**Authors:** Jiawen Wang, Yanping Liu, Han Zhu, Kourong Miao

**Affiliations:** Hematology, The First Affiliated Hospital with Nanjing Medical University, Nanjing, 210029 China

**Keywords:** Allogeneic hematopoietic stem cell transplantation, Unrelated donors mismatch, Survival

## Abstract

Allogeneic hematopoietic stem cell transplantation (allo-HSCT) is an important treatment option for hematologic diseases. However, limited studies have evaluated the prognosis of patients receiving single human leukocyte antigen (HLA) mismatched unrelated donor allo-HSCT (HLA 9/10 MMUD-HSCT) compared to those receiving fully matched unrelated donor allo-HSCT (10/10 MUD-HSCT). This study retrospectively analyzed 126 cases of unrelated donor allo-HSCT (URD-HSCT) at our center, in which anti-human thymocyte globulin (ATG, 7.5 mg/kg) was used as a graft-versus-host disease (GVHD) prophylaxis strategy. The MUD-HSCT group had a significantly lower incidence of grade II–IV acute GVHD (13.89% vs. 35.19% in the MMUD-HSCT group, *p* = 0.005). In contrast, the incidence of moderate-to-severe chronic GVHD (cGVHD) did not differ significantly between the two groups (16.67% vs. 29.63%, *p* = 0.083). The median follow-up time was 16.98 months (range: 7.88–38.55). There were no significant differences between the two groups in the 1-year cumulative incidence of relapse (CIR) (*p* = 0.707), 3-year CIR (*p* = 0.764), 1-year disease-free survival (DFS) (*p* = 0.954), 3-year DFS (*p* = 0.888), 1-year overall survival (OS) (*p* = 0.611), 3-year OS (*p* = 0.796), 3-year non-relapse mortality (NRM) (*p* = 0.711), or GVHD-free relapse-free survival (GRFS) (*p* = 0.546). The estimated median OS and DFS times were not reached in either group. In conclusion, under an ATG-based GVHD prophylaxis regimen, HLA 9/10 MMUD-HSCT is a viable alternative donor option, offering comparable clinical outcomes to those of fully matched unrelated donor HSCT.

## Introduction

Allogeneic hematopoietic stem cell transplantation (allo-HSCT) plays a critical role in the treatment of many malignant hematological diseases and, in some cases, may offer the only potential cure. For patients undergoing HSCT, a matched sibling donor (MSD) is generally preferred, as it is associated with lower risks of graft-versus-host disease (GVHD) and non-relapse mortality (NRM). However, MSDs are not always available. Their availability varies considerably depending on the patient’s ethnicity and age, with matching rates ranging from 13 to 51% [1]. In recent years, ongoing advancements in transplantation techniques have established unrelated donors (URDs) as an important alternative to MSDs. Including mismatched unrelated donors (MMUDs) in the donor pool can increase the matching rate to approximately 82%–86% [2]. In 2023, approximately 13% of patients with malignant hematological diseases who underwent transplantation received grafts from URDs. Of these, 1,069 patients with acute myeloid leukemia (AML) underwent URD allo-HSCT (URD-HSCT), accounting for 10% of all transplant recipients. Similarly, 628 patients with acute lymphoblastic leukemia (ALL) received URD-HSCT (10%), and 330 patients with myelodysplastic syndrome (MDS) received transplants from URDs, representing 12% of all hematopoietic stem cell transplants performed [3]. The use of URD-HSCT has expanded access to transplantation, thereby improving the prognosis for patients with malignant hematological conditions. However, there is limited research comparing the clinical outcomes of fully matched unrelated donor allo-HSCT (MUD-HSCT) with those of single human leukocyte antigen (HLA) mismatched unrelated donor allo-HSCT (HLA 9/10 MMUD-HSCT). Therefore, this study aimed to evaluate whether clinical outcomes differ between HLA 9/10 MMUD-HSCT and MUD-HSCT by analyzing URD-HSCT data from our center.

## Patients and methods

### Patients

A total of 126 patients who underwent allogeneic stem cell transplantation at the Department of Hematology, Jiangsu Province Hospital, Nanjing, Jiangsu Province, People’s Republic of China, between 2017 and 2025 were retrospectively analyzed in this study. Follow-up was conducted through outpatient visits and telephone calls, with the last follow-up date being January 8, 2025.

### High-resolution HLA typing methods

High-resolution HLA typing was performed for at least ten loci, including HLA-A, -B, -C, -DRB1, and -DQB1. Typing reagents included One Lambda, TBG, and CareDx AlloSeq Tx17, with detection methods using either next-generation sequencing (NGS) or sequencing-based typing (SBT).

### Conditioning regimens and GVHD prevention

All enrolled patients were eligible for myeloablative conditioning regimens. The standard Busulfan/Cyclophosphamide (Bu/Cy) regimen consisted of Busulfan 3.2 mg/kg/day from day −7 to day −4, and Cyclophosphamide 60 mg/kg/day from day −3 to day −2. Alternatively, the Fludarabine/Busulfan (Flu/Bu) regimen included Fludarabine 30 mg/m^2^/day from day −6 to day −2, and Busulfan 3.2 mg/kg/day from day −5 to day −3. Total body irradiation was not used in any of the conditioning regimens. Stem cells were infused intravenously on day 0. The minimum total nucleated cell (TNC) dose was 5 × 10^8^/kg, and the minimum CD34 + cell dose was 2 × 10^6^/kg.

The GVHD prophylaxis regimen for URD-HSCT patients included Cyclosporin A (CsA), short-course Methotrexate (MTX), anti-thymocyte globulin (ATG; Sanofi, France), and Mycophenolate Mofetil (MMF). MTX was administered at 15 mg/m^2^ on day 1, and 10 mg/m^2^ on days 3, 6, and 11. CsA serum trough levels were monitored twice weekly and maintained between 200–400 ng/mL. ATG was given at 2.5 mg/kg/day from day −4 to day −2. Oral MMF was administered at 500 mg twice daily starting on day −1 and was tapered after 28 days in the absence of acute GVHD (aGVHD). Acute GVHD was graded according to the criteria of the MAGIC consortium [4], and chronic GVHD (cGVHD) was graded following the NIH Consensus Conference guidelines [5].

### Post-transplant monitoring

Bone marrow aspiration was routinely performed on days 30, 60, 90, 180, 270, and 360 after HSCT. At each time point, 2 mL of bone marrow was collected in ethylenediaminetetraacetic acid (EDTA) anticoagulant tubes for the detection of minimal residual disease (MRD) by flow cytometry in patients with AML and ALL. Flow cytometry was conducted using fluorescently labeled antibodies supplied by BD Biosciences (Becton Dickinson, Franklin Lakes, NJ, USA). Hematopoietic chimerism was assessed by fluorescence in situ hybridization (FISH) for sex chromosome determination in sex-mismatched donor-recipient pairs, or by short tandem repeat (STR) analysis via polymerase chain reaction (PCR) in same-sex pairs. In patients with high-risk gene mutations, targeted therapies were used for post-transplant maintenance. If MRD positivity was detected after transplant, low-dose azacitidine was administered.

Cytomegalovirus (CMV) and Epstein-Barr virus (EBV) reactivation were defined as two consecutive positive plasma samples with a viral load ≥ 500 copies/mL. Quantitative polymerase chain reaction (qPCR) testing for CMV and EBV was performed once or twice weekly until day 100 post-transplant. Continued monitoring was guided by clinical symptoms. Letermovir was routinely administered until day 100 post-transplant, while Valacyclovir and Trimethoprim/Sulfamethoxazole were given until one year post-transplant. Cephalosporins were generally used for antibacterial prophylaxis.

Post-transplant, patients were monitored daily for blood pressure, oxygen saturation, and immunoglobulin levels. Intravenous immunoglobulin was administered if levels were found to be low. Pulmonary function was assessed every six months following transplantation. For pneumocystis pneumonia prophylaxis, Trimethoprim/Sulfamethoxazole was taken every Tuesday and Friday.

### Supportive care

During the transplantation period, patients were housed in laminar flow rooms to ensure protective isolation. All patients received prophylactic antifungal, antibacterial, and antiviral treatments to prevent infections. If patients entered a neutropenic state or developed an infection, the antimicrobial regimen was promptly escalated. To prevent hepatic veno-occlusive disease, all recipients received prostaglandin E1 and reduced glutathione during the transplant process. In the Bu/Cy conditioning regimen, Mesna was administered at 120 mg/kg/day from day 3 to day 2 to prevent urotoxicity. In the Flu/Bu regimen, valproic acid was administered at a dose of 0.5 g every 8 h to prevent neurotoxicity. Daily monitoring of complete blood counts, liver and kidney function, and electrolytes was conducted throughout the transplantation period. Blood products were transfused as needed, with the aim of maintaining hemoglobin levels above 70 g/L and platelet counts above 20 × 10^9^/L. Beginning on day 5 post-transplant, recombinant human granulocyte colony-stimulating factor and recombinant human thrombopoietin were routinely administered to promote hematopoietic recovery.

### Definitions

High-resolution HLA typing was performed on all samples, covering 10 HLA loci: HLA-A, -B, -C, -DRB1, and -DQB1. HLA 9/10 MMUD refers to a single-locus mismatch between the donor and recipient. Relapse of ALL, AML, and MDS was defined as the presence of ≥ 5% blasts in the bone marrow, the appearance of blasts in peripheral blood, or evidence of extramedullary disease, respectively. Relapse of multiple myeloma (MM) was defined as the reappearance of M-protein in the blood or urine, confirmed by immunofixation electrophoresis, or a bone marrow plasma cell percentage of ≥ 5%. Relapse of chronic myeloid leukemia (CML) was defined by the detection of the BCR-ABL fusion gene. Relapse of NK/T-cell lymphoma was assessed through imaging studies and bone marrow examination. Neutrophil engraftment was defined as an absolute neutrophil count ≥ 0.5 × 10^9^/L for three consecutive days. Platelet engraftment was defined as a platelet count ≥ 20 × 10^9^/L for seven consecutive days without transfusion support. Overall survival (OS) was defined as the time from stem cell infusion to death or last follow-up. Disease-free survival (DFS) was defined as the time from stem cell infusion to relapse, death from any cause, or last follow-up. Non-relapse mortality (NRM) was defined as death from any cause other than relapse of the primary disease. GVHD-free**,** relapse-free survival (GRFS) was defined as survival without grade III–IV aGVHD, cGVHD requiring systemic therapy, relapse, or death. A complex karyotype was defined as the presence of three or more chromosomal abnormalities. MRD was considered positive (MRD⁺) if ≥ 0.01%, and negative (MRD⁻) if < 0.01%. Complete chimerism (CC) was defined as a donor chimerism rate of ≥ 95%. All other cases were classified as incomplete chimerism.

#### Statistical methods

Cumulative incidence of relapse (CIR), OS, NRM, and DFS were calculated using the Kaplan–Meier method. Chi-square tests were used to compare categorical variables, while non-parametric Mann–Whitney U tests were applied to assess differences in median values for continuous variables. All statistical analyses were performed using R version 4.3.0 and SPSS version 25.

## Results

### Total patients

#### Clinical characteristics

This study included 126 patients with malignant hematological diseases. The clinical characteristics of patients in the MUD-HSCT group and the HLA 9/10 MMUD-HSCT group are summarized in Table 1. Of the 126 patients, 74 were male (58.73%) and 52 were female (41.27%). The underlying diseases included AML in 46 patients (36.51%), ALL in 33 (26.19%), MDS in 24 (19.05%), and other hematologic malignancies in 23 (18.25%), including MM, NK/T-cell lymphoma, and CML. The median age at transplantation was 43.50 years (range: 32.25–52.00), and the median follow-up duration was 16.98 months (range: 7.88–38.55). The median white blood cell count at initial diagnosis was 4.54 × 10⁹/L (range: 2.39–15.54), and the median time from diagnosis to transplantation was 6.35 months (range: 4.67–9.79). There were no statistically significant differences between the MUD-HSCT and HLA 9/10 MMUD-HSCT groups in terms of gender distribution, age, initial white blood cell count, pre-transplant disease status, or disease type (*p* > 0.05).

**Table 1:** Baseline characteristics of MUD-HSCT group and HLA 9/10 MMUD-HSCT group before transplantation

Variables	Total patients (*n* = 126)	MUD-HSCT (*n* = 72)	MMUD-HSCT (*n* = 54)	Statistic	*P*-value
Age at transplantation, median (range), years	44 (32, 52)	44 (30, 53)	44 (35, 51)	Z = −0.06	0.949
WBC at diagnosis, median (range), 10⁹/L	4.54 (2.39, 15.54)	3.58 (2.35, 15.32)	6.75 (2.74, 19.63)	Z = −0.99	0.320
Time from diagnosis to transplantation, median (range), months	6.35 (4.67, 9.79)	6.50 (4.93, 9.61)	5.82 (4.47, 9.82)	Z = −0.66	0.509
Gender				χ^2^ = 2.97	0.085
Male	74 (58.73)	47 (65.28)	27 (50.00)		
Female	52 (41.27)	25 (34.72)	27 (50.00)		
Disease				χ^2^ = 1.77	0.621
AML	46 (36.51)	25 (34.72)	21 (38.89)		
ALL	33 (26.19)	18 (25.00)	15 (27.78)		
Others	23 (18.25)	16 (22.22)	7 (12.96)		
MDS	24 (19.05)	13 (18.06)	11 (20.37)		
Disease status before transplantation				χ^2^ = 0.69	0.407
CR and MRD (-)	86 (6825)	47 (65.26)	25 (62.50)		
PR/NR or MRD (+)	40 (31.75)	25 (34.72)	15 (37.50)		

*Abbreviations*: *AML* Acute myeloid leukemia, *ALL* Acute lymphoblastic leukemia, *MDS* Myelodysplastic syndrome, *MRD* Minimal Residual Disease, *CR* Complete remission, *PR/NR* Partial remission/No remission, *HSCT* Hematopoietic Stem Cell Transplantation, *MUD* Matched Unrelated Donor, *MMUD* Mismatched Unrelated Donor. MRD < 0.01% was considered as (MRD −)

#### Transplantation characteristics

Of the 126 patients, 72 (57.14%) received MUD-HSCT and 54 (42.86%) received HLA 9/10 MMUD-HSCT. There were no significant differences between the two groups in terms of conditioning regimens, gender matching, blood type compatibility, infused mononuclear cell (MNC) counts, or infused CD34⁺ cell counts (*p* > 0.05) (Table 2).

**Table 2:** Transplantation characteristics of MUD-HSCT group and HLA 9/10 MMUD-HSCT group before transplantation

Variables	Total patients (*n* = 126)	MUD-HSCT (*n* = 72)	MMUD-HSCT (*n* = 54)	Statistic	*P*-value
MNC count, median (range), 10^8^/kg	8.62 (6.85, 11.16)	8.29 (6.35, 11.56)	8.80 (7.20, 11.06)	Z = −0.46	0.647
CD34 + count, median (range), 10^6^/kg	6.50 (4.12, 9.59)	6.71 (3.83, 8.93)	6.44 (4.57, 10.39)	Z = −0.80	0.422
Gender matching				χ^2^ = 0.18	0.675
Matched	75 (59.52)	44 (61.11)	31 (57.41)		
Mismatched	51 (40.48)	28 (38.89)	23 (42.59)		
ABO compatibility				χ^2^ = 0.68	0.410
Compatible	59 (46.83)	36 (50.00)	23 (42.59)		
Incompatible	67 (53.17)	36 (50.00)	31 (57.41)		
Conditioning regimen				χ^2^ = 0.38	0.537
Bu/Cy	60 (47.62)	36 (50.00)	24 (44.44)		
Flu/Bu	66 (52.38)	36 (50.00)	30 (55.56)		

*Abbreviations*: *MNC* Mononuclear cell, *Bu/Cy* Busulfan/Cyclophosphamide, *Flu/Bu* Fludarabine/Busulfan, *HSCT* Hematopoietic Stem Cell Transplantation, *MUD* Matched Unrelated Donor, *MMUD* Mismatched Unrelated Donor

## Transplantation outcomes in MUD-HSCT and HLA 9/10 MMUD-HSCT groups

### Engraftment

Among all patients, 124 achieved platelet engraftment within 28 days post-transplantation, resulting in an overall engraftment rate of 98.41%. The median time to platelet engraftment was 12.00 days (range: 11.00–13.00). In the MUD-HSCT group, the median time to platelet engraftment was 12.00 days (11.00–14.00), compared to 11.00 days (11.00–12.00) in the HLA 9/10 MMUD-HSCT group (Table 3). Neutrophil engraftment was also achieved in 124 patients within 28 days. One patient in the HLA 9/10 MMUD-HSCT group failed to achieve neutrophil engraftment, and another died before engraftment, resulting in a neutrophil engraftment rate of 98.41%. The overall median time to neutrophil engraftment was 11.00 days (range: 11.00–12.00). In the MUD-HSCT group, the median time was 11.00 days (11.00–13.00), and in the HLA 9/10 MMUD-HSCT group, it was 11.00 days (11.00–11.00). There were no statistically significant differences between the two groups in the rates or median times of platelet and neutrophil engraftment (*p* > 0.05). The CC rate within one month post-transplantation was 98.41%, with both the MUD-HSCT and HLA 9/10 MMUD-HSCT groups showing a rate of 99.21%. At three years post-transplant, the CC rate was 47.6% overall, with 72.22% in the MUD-HSCT group and 77.78% in the HLA 9/10 MMUD-HSCT group. These differences were not statistically significant (*p* > 0.05).

**Table 3:** Transplantation outcomes between the MUD-HSCT group and the MMUD-HSCT group

Variables	Total patients (*n* = 126)	MUD-HSCT (*n* = 72)	MMUD-HSCT (*n* = 54)	Statistic	*P*-value
Time to Neutrophil Engraftment, Median (range), Days	11.00 (11.00, 12.00)	11.00 (11.00, 13.00)	11.00 (11.00, 11.00)	Z = −1.42	0.156
Time to Megakaryocyte Engraftment, Median (range), Days	12.00 (11.00, 13.00)	12.00 (11.00, 14.00)	11.00 (11.00, 12.00)	Z = −1.61	0.107
Post-Transplant MRD				χ^2^ = 0.90	0.343
Negative	87 (69.60)	47 (66.20)	40 (74.07)		
Positive	38 (30.40)	24 (33.80)	14 (25.93)		
Hemorrhagic cystitis				χ^2^ = 2.46	0.117
No	74 (58.73)	38 (52.78)	36 (66.67)		
Yes	52 (41.27)	34 (47.22)	18 (33.33)		
CMV reactivation				χ^2^ = 0.25	0.617
No	87 (69.05)	51 (70.83)	36 (66.67)		
Yes	39 (30.95)	21 (29.17)	18 (33.33)		
EBV reactivation				χ^2^ = 0.22	0.638
No	52 (41.27)	31 (43.06)	21 (38.89)		
Yes	74 (58.73)	41 (56.94)	33 (61.11)		
TA-TMA					
No	125 ()	72	53		
Yes	1 ()	0	1		
aGVHD				χ^2^ = 0.04	0.837
No	62 (49.21)	36 (50.00)	26 (48.15)		
Yes	64 (50.79)	36 (50.00)	28 (51.85)		
Grade II-IV aGVHD				χ^2^ = 7.90	**0.005**
No	97 (76.98)	62 (86.11)	35 (64.81)		
Yes	29 (23.02)	10 (13.89)	19 (35.19)		
cGVHD				χ^2^ = 0.00	0.959
No	58 (46.03)	33 (45.83)	25 (46.30)		
Yes	68 (53.97)	39 (54.17)	29 (53.70)		
Moderate-severe cGVHD				χ^2^ = 3.00	0.083
No	98 (76.98)	60 (83.33)	38 (70.37)		
Yes	28 (22.22)	12 (16.67)	16 (29.63)		
GRFS				χ^2^ = 0.36	0.546
No	41 (32.54)	25 (34.72)	16 (29.63)		
Yes	85 (67.46)	47 (65.28)	38 (70.37)		
Relapse				χ^2^ = 0.41	0.524
No	92 (73.02)	51 (70.83)	41 (75.93)		
Yes	34 (26.98)	21 (29.17)	13 (24.07)		
Mortality in 100 Days				χ^2^ = 0.00	1.000
No	123 (97.62)	70 (97.22)	53 (98.15)		
Yes	3 (2.38)	2 (2.78)	1 (1.85)		
Complete Chimerism in 3 years				χ^2^ = 0.50	0.478
No	32 (25.40)	20 (27.78)	12 (22.22)		
Yes	94 (74.60)	52 (72.22)	42 (77.78)		
NRM				χ^2^ = 0.00	1.000
No	120 (95.24)	69 (95.83)	51 (94.44)		
Yes	6 (4.76)	3 (4.17)	3 (5.56)		
Death				χ^2^ = 0.01	0.905
No	95 (75.40)	54 (75.00)	41 (75.93)		
Yes	31 (24.60)	18 (25.00)	13 (24.07)		

*Abbreviations MRD* Measurable residual disease, *CMV* Cytomegalovirus, *EBV* Epstein-Barr virus, *aGVHD* Acute Graft-Versus-Host Disease, *cGVHD* Chronic Graft-Versus-Host Disease, *GRFS* GVHD-free relapse-free survival, *NRM* No relapse mortality, *HSCT* Hematopoietic Stem Cell Transplantation, *MUD* Matched Unrelated Donor, *MMUD* Mismatched Unrelated Donor

### Relapse and NRM

**Fig. 1:**
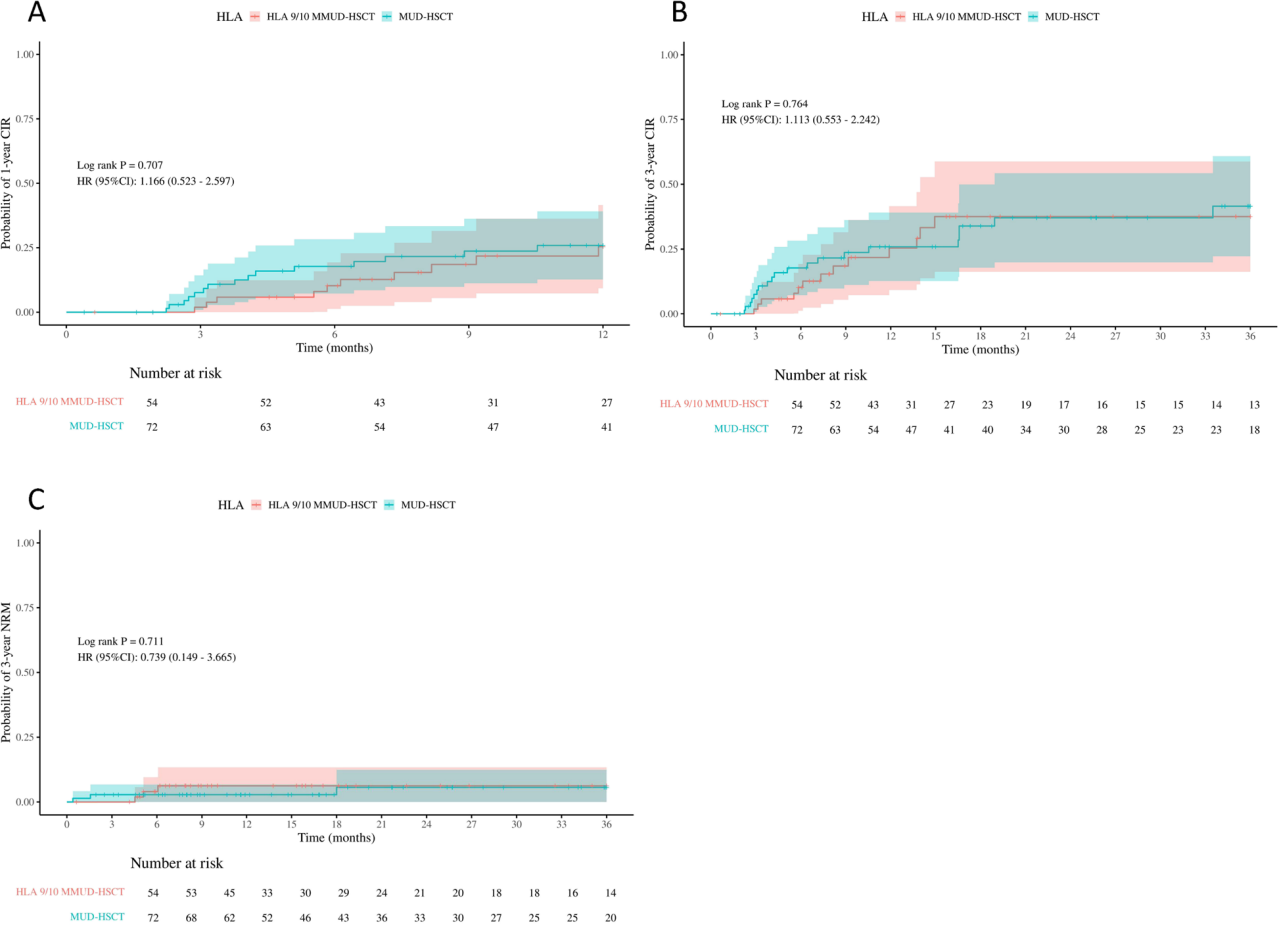
The probability of 1-year cumulative incidence of relapse (CIR), 3-year CIR and 3-year no relapse mortality (NRM) in transplantation patients in MUD-HSCT and HLA 9/10 MMUD-HSCT (**A**-**C**)

**Fig. 2:**
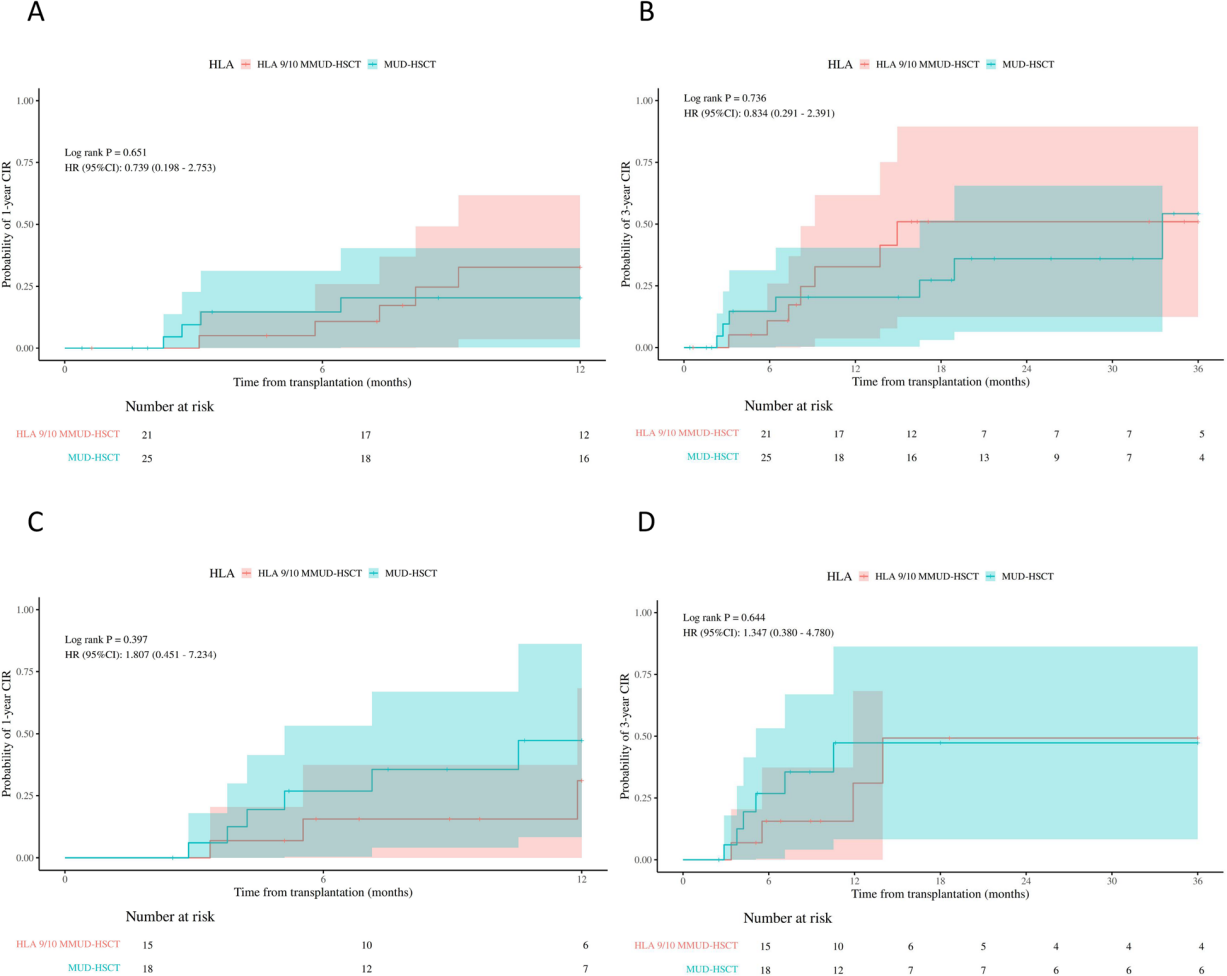
The probability of 1-year cumulative incidence of relapse (CIR), 3-year CIR in acute myeloid leukemia (**A**-**B**) and acute lymphoblastic leukemia (**C**-**D**) patients in MUD-HSCT and HLA 9/10 MMUD-HSCT (**A-B**)

A total of 34 patients (26.98%) experienced disease relapse, including 21 patients (29.17%) in the MUD-HSCT group and 13 patients (24.07%) in the HLA 9/10 MMUD-HSCT group. There were no significant differences between the two groups in the 1-year CIR, which was 22.82% in the MUD-HSCT group and 22.44% in the HLA 9/10 MMUD-HSCT group (*p* = 0.707), nor in the 3-year CIR (33.98% vs. 31.29%, *p* = 0.764). A total of 6 patients (4.76%) experienced non-relapse mortality, including three patients (4.17%) in the MUD-HSCT group and three patients (5.56%) in the HLA 9/10 MMUD-HSCT group. The 3-year cumulative NRM rates were comparable between the two groups (5.48% in the MUD-HSCT group vs. 6.06% in the HLA 9/10 MMUD-HSCT group, *p* = 0.711) (Fig. 1A-C). Similarly, within the AML and ALL subgroups, no significant differences were observed in the 1-year or 3-year CIR between the two transplant groups (Fig. 2).

### DFS and OS

**Fig. 3:**
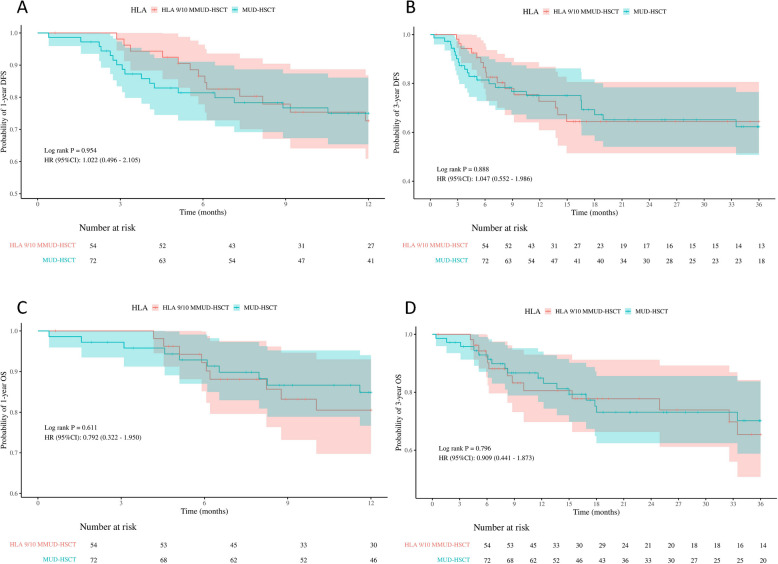
The probability of 1-year disease free survival (DFS), 3-year DFS, 1-year overall survival (OS) and 3-year OS in transplantation patients in MUD-HSCT and HLA 9/10 MMUD-HSCT (**A**-**D**)

**Fig. 4:**
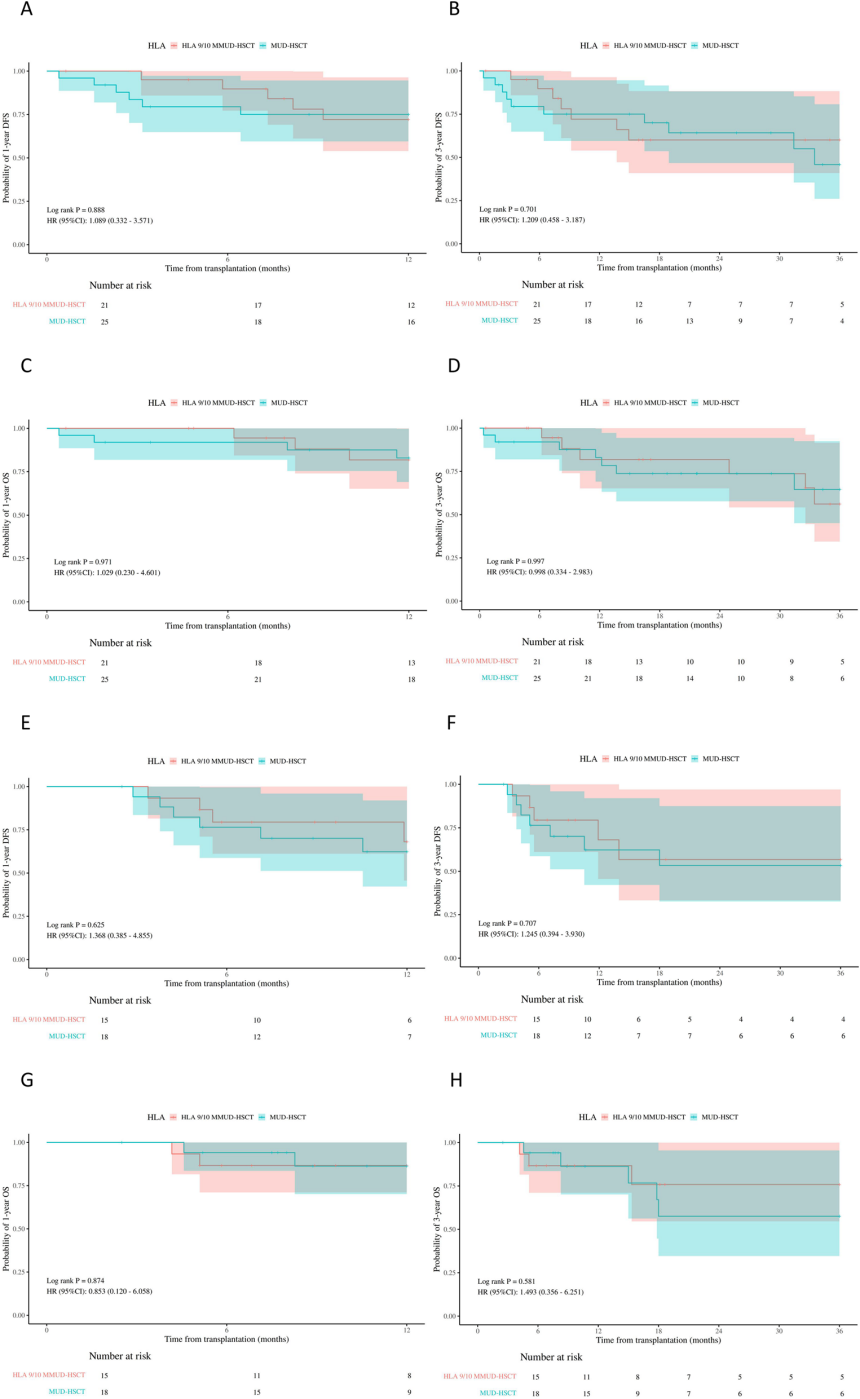
The probability of 1-year disease free survival (DFS), 3-year DFS, 1-year overall survival (OS), 3-year OS in acute myeloid leukemia (**A**-**D**) and acute lymphoblastic leukemia (**E**-**H**) patients in MUD-HSCT and HLA 9/10 MMUD-HSCT

A total of 31 patients (24.60%) died, including 18 patients (25.00%) in the MUD-HSCT group and 13 patients (24.07%) in the HLA 9/10 MMUD-HSCT group. The overall mortality rate within 100 days post-transplant was 2.38%, with rates of 2.78% in the MUD-HSCT group and 1.85% in the HLA 9/10 MMUD-HSCT group. There were no significant differences in the 1-year DFS rates (75.04% for the MUD-HSCT group vs. 72.69% for the HLA 9/10 MMUD-HSCT group, *p* = 0.954) or the 3-year DFS rates (62.30% vs. 64.40%, *p* = 0.888). Similarly, no significant differences were observed in the 1-year OS rates (84.90% vs. 80.54%, *p* = 0.611) or the 3-year OS rates (70.18% vs. 65.41%, *p* = 0.796) between the two groups. The median OS and DFS times were not reached in either group (Fig. 3A-D). In subgroup analysis of AML and ALL patients, no differences were found in 1-year or 3-year DFS and OS between the MUD-HSCT and HLA 9/10 MMUD-HSCT groups. (Fig. 4).

### Post-transplant complications

**Fig. 5:**
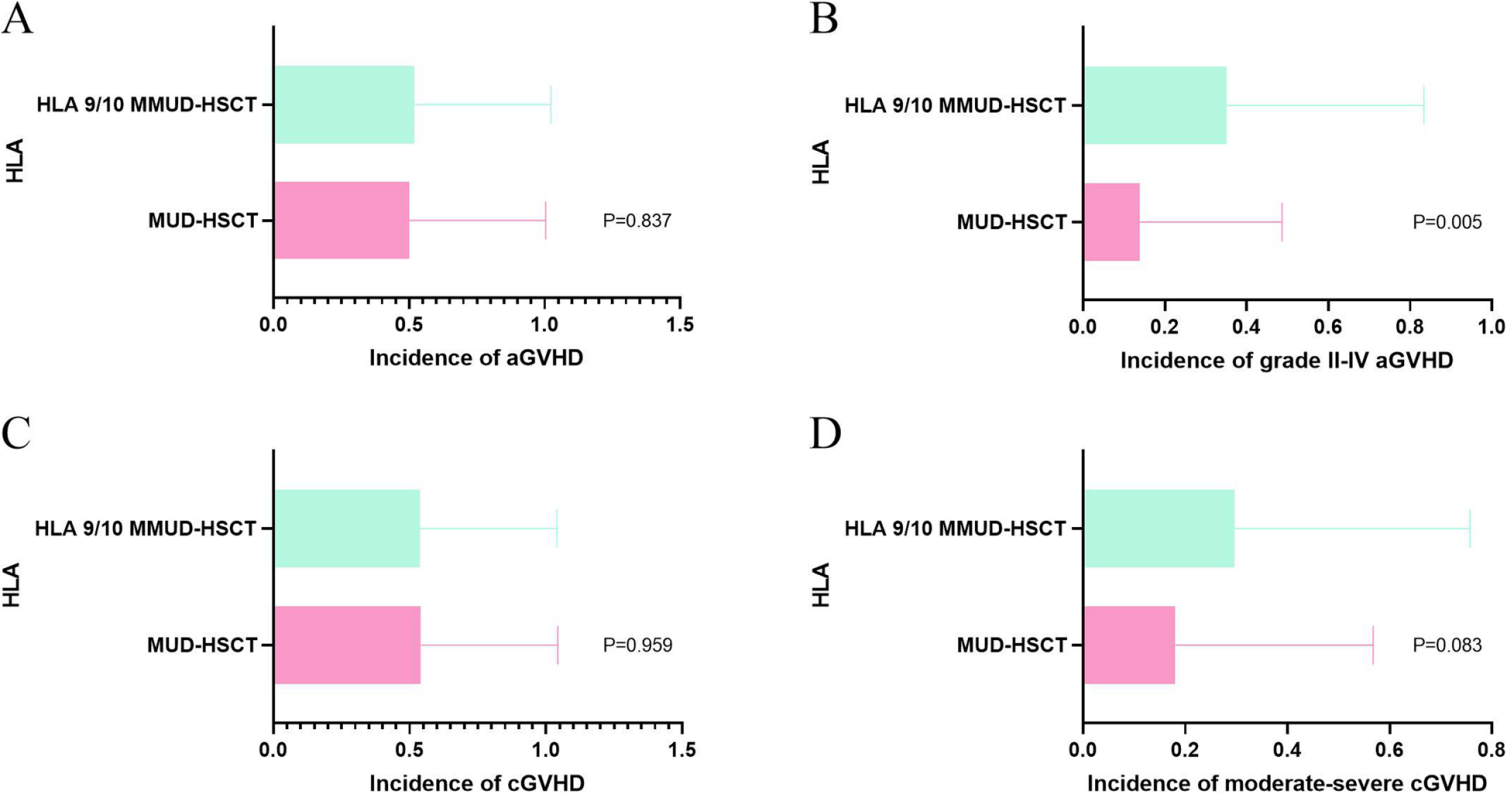
The incidence of acute graft-versus-host disease (aGVHD), grade II-IV aGVHD, chronic GVHD (cGVHD), moderate-severe cGVHD transplantation patients in MUD-HSCT and HLA 9/10 MMUD-HSCT (**A**-**D**)

A total of 64 patients (50.79%) developed aGVHD, including 36 patients (50.00%) in the MUD-HSCT group and 28 patients (51.85%) in the HLA 9/10 MMUD-HSCT group. There was no significant difference in the overall incidence of aGVHD between the two groups (*p* = 0.837). Among these, 29 patients (23.02%) experienced grade II–IV aGVHD, with a significantly higher incidence observed in the HLA 9/10 MMUD-HSCT group (19 patients, 35.19%) compared to the MUD-HSCT group (10 patients, 13.89%) (*p* = 0.005). (Fig. 5A-B).

cGVHD occurred in 68 patients (53.97%), including 39 patients (54.17%) in the MUD-HSCT group and 29 patients (53.70%) in the HLA 9/10 MMUD-HSCT group. The difference in cGVHD incidence between the groups was not statistically significant (*p* = 0.959). Among these, 28 patients (22.22%) developed moderate to severe cGVHD—12 patients (16.67%) in the MUD-HSCT group and 16 patients (29.63%) in the HLA 9/10 MMUD-HSCT group. Although the incidence was higher in the HLA 9/10 MMUD-HSCT group, the difference did not reach statistical significance (*p* = 0.083). (Fig. 5C-D).

GRFS was achieved in 85 patients (67.46%), including 47 patients (65.28%) in the MUD-HSCT group and 38 patients (70.37%) in the HLA 9/10 MMUD-HSCT group, with no significant difference between the groups (*p* = 0.546). Additionally, there were no statistically significant differences between the two groups in the incidence of hemorrhagic cystitis, CMV reactivation, or EBV reactivation (*p* > 0.05). No cases of hepatic veno-occlusive disease or transplant-associated thrombotic microangiopathy were observed in either group.

## Discussion

With ongoing advancements in transplantation technology, URD-HSCT has become an important alternative to MSD-HSCT. Although numerous studies have reported that URD-HSCT is associated with a higher risk of NRM and GVHD compared to MSD-HSCT, this risk increases with the number of HLA mismatches [6]. One study involving 411 children with high-risk ALL found no significant differences between MUD-HSCT and MSD-HSCT in 4-year event-free survival (67% ± 3% vs. 71% ± 5%, *p* = 0.405), overall survival (73% ± 3% vs. 79% ± 4%, *p* = 0.230), or cumulative incidence of relapse (22% ± 2% vs. 24% ± 4%, *p* = 0.732) [7]. The Center for International Blood and Marrow Transplant Research (CIBMTR) reported on outcomes in over 25,000 patients who underwent allo-HSCT, evaluating the effectiveness of alternative donors by comparing 1-year OS rates against the baseline of MSD-HSCT. The odds ratio for 1-year OS was 0.87 (95% CI: 0.78–0.97, *p* = 0.010) for MUD-HSCT; 0.76 (95% CI: 0.68–0.84, *p* < 0.001) for haploidentical donor HSCT; 0.69 (95% CI: 0.59–0.81, *p* < 0.001) for HLA 7/8 MMUD-HSCT; and 0.50 (95% CI: 0.39–0.64, *p* < 0.001) for umbilical cord blood HSCT. These real-world data highlight a clear hierarchy in donor selection, with MSD being the preferred option, followed by MUD, and finally MMUD [8].

The number of mismatched HLA loci is directly associated with the risk of GVHD and NRM. Studies have shown that, compared to fully matched (HLA 8/8) MUD-HSCT, an increasing number of mismatched HLA loci correlates with higher rates of NRM, grade II–IV aGVHD, and transplant-related mortality, although relapse and overall aGVHD incidence are not significantly associated with the number of HLA matches [2, 9]. For patients without MSD or HID, MMUD remains a viable option. A study involving 3,336 MMUD-HSCT recipients demonstrated acceptable toxicity and favorable outcomes, with a 1-year OS rate of 63.9% (95% CI: 0.57–0.71) and a 3-year OS rate of 42.1% (95% CI: 0.34–0.50). The incidences of grade II-IV aGVHD and cGVHD were 36.4% (95% CI 0.31–0.42) and 41.2% (95% CI 0.35–0.48), respectively. After a median follow-up of 2.6 years (range: 1–5), the NRM was 22.6% (95% CI 0.17–0.29). Recently, the focus has shifted to prioritizing high-resolution matching at specific HLA loci—primarily HLA-A, -B, -C, and -DRB1. An analysis of 3,857 URD-HSCT patients who underwent myeloablative conditioning showed that high-resolution matching at these loci was associated with the highest survival rates. Notably, a single mismatch at HLA-DQB1 did not significantly impact outcomes, suggesting that HLA 10/10 and HLA 9/10 matches (excluding DQB1) offer comparable survival, reinforcing the predictive value of matching at the four primary loci [10–13]. GVHD remains a major concern in URD-HSCT, as severe GVHD substantially increases NRM risk. Therefore, effective GVHD prophylaxis is critical. Currently, clinical strategies include ATG, and post-transplant cyclophosphamide (PTCY). One study found that compared to higher doses of ATG-Genzyme (≥ 7.5 mg/kg), ATG-Fresenius (20 mg/kg) was associated with significantly higher rates of cGVHD (HR 3.14; 95% CI 1.53–6.45; *p* = 0.002) and moderate-to-severe cGVHD (HR 2.92; 95% CI 1.23–6.92; *p* = 0.015). However, no significant differences in aGVHD or cGVHD incidence were observed between higher-dose and lower-dose ATG-Genzyme (≤ 6 mg/kg) groups [14]. Some studies have suggested that PTCY may offer superior GVHD prevention, showing lower cumulative incidences of grade II–IV and III–IV aGVHD at 100 days, lower cGVHD and relapse rates [15] and higher GVHD-free survival, progression-free survival, and OS compared to ATG [16–18]. However, other reports found no significant differences between ATG and PTCY in 2-year rates of moderate-to-severe cGVHD, CIR, OS, or NRM [15, 19]. A randomized multicenter phase II trial comparing ATG (2.5 mg/kg on days –2 and –1) and PTCY (50 mg/kg on days + 3 and + 4) under non-myeloablative conditioning found no significant differences in GVHD, GRFS, DFS, CIR, NRM, or OS at 12 months post-HSCT. The 12-month GRFS was 54.5% in the PTCY group and 43.2% in the ATG group (*p* = 0.27) [20]. Recently, combination strategies incorporating both ATG and PTCY have been explored. These dual prophylactic approaches have been shown to reduce the incidence of grade III–IV aGVHD and cGVHD, while improving survival, especially GVHD-free, relapse-free survival [21, 22]. In our study, under the ATG-based GVHD prophylaxis regimen (7.5 mg/kg), there were no significant differences in the overall incidence of aGVHD and cGVHD between the MUD-HSCT and HLA 9/10 MMUD-HSCT groups. However, the incidence of grade II–III aGVHD was higher in the HLA 9/10 MMUD-HSCT group, suggesting the need for further research into optimal GVHD prevention strategies and ATG dosing in this population. It is important to note that all patients at our center received only a higher dose of ATG, without exploration of lower doses. Thus, future studies are needed to assess the clinical outcomes of patients receiving lower-dose ATG regimens. The relatively low NRM observed in this study may be attributed to the fact that most deaths were relapse-related, with fewer deaths due to non-relapse causes. This may reflect the high level of medical accessibility at our center. Post-transplant, most patients rent accommodations near the hospital for at least three months, enabling rapid access to outpatient or emergency care when needed. Consequently, the post-transplant infection rate in our ward is exceptionally low. Although GVHD remains a concern, the incidence of severe GVHD is relatively low. Furthermore, patients who develop grade II–IV GVHD are quickly readmitted to the transplant ward and receive immediate, standardized treatment.

## Conclusion

This study demonstrates that under an ATG-containing in vivo T cell depletion protocol, HLA 9/10 MMUD-HSCT and MUD-HSCT yield comparable clinical outcomes. These findings suggest that HLA 9/10 MMUD-HSCT is a viable alternative donor option, significantly broadening the pool of eligible transplant donors and enabling more patients with malignant hematological diseases to benefit from HSCT.

## Data Availability

No datasets were generated or analysed during the current study.
